# Investigating Different Clinical Manifestations of *Staphylococcus aureus* Infections in Childhood—Can D-Dimer and Fibrinogen Predict Deep Tissue Invasion?

**DOI:** 10.3390/children12080959

**Published:** 2025-07-22

**Authors:** Pınar Önal, Gözde Apaydın Sever, Beste Akdeniz Eren, Gülşen Kes, Ayşe Ayzıt Kılınç Sakallı, Fatih Aygün, Gökhan Aygün, Haluk Çokuğraş, Fatma Deniz Aygün

**Affiliations:** 1 Department of Pediatric Infectious Diseases, Cerrahpaşa Faculty of Medicine, Istanbul University-Cerrahpasa, Istanbul 34303, Turkey; gozde_apaydin@hotmail.com (G.A.S.); besteak.90@gmail.com (B.A.E.); gulsen.kes.17@gmail.com (G.K.); cokugras@gmail.com (H.Ç.); fdenizaygun@gmail.com (F.D.A.); 2 Department of Pediatric Pulmonology, Cerrahpaşa Faculty of Medicine, Istanbul University-Cerrahpasa, Istanbul 34303, Turkey; ayse.kilinc@iuc.edu.tr; 3 Department of Pediatric Intensive Care, Cerrahpaşa Faculty of Medicine, Istanbul University-Cerrahpasa, Istanbul 34098, Turkey; faygun9@hotmail.com; 4 Department of Medical Microbiology, Cerrahpaşa Faculty of Medicine, İstanbul University-Cerrahpaşa, Istanbul 34098, Turkey; gokhan.aygun@iuc.edu.tr

**Keywords:** *Staphylococcus aureus*, infection, children, deep-seated

## Abstract

**Background**: *Staphylococcus aureus* is a significant pathogen causing both local and systemic infections in children, with deep tissue involvement leading to severe complications. This study aimed to assess clinical manifestations and identify risk factors for deep tissue involvement in pediatric *S. aureus* infections. **Methods:** All children between 1 month and 18 years who had *S. aureus* growth in blood, pus, or joint fluid culture were included. **Results**: A total of 61 patients (median age 55 months) were included, with 22.9% having deep tissue infections. Osteoarticular infections, pyomyositis, and pulmonary involvement were common. Deep-seated infections were significantly associated with community-acquired infections and positive hemocultures after 72 h (*p* < 0.01). Laboratory results showed significantly higher levels of C-reactive protein, sedimentation rate, D-dimer, and fibrinogen in the group with deep-seated infections (*p* = 0.02, *p* = 0.018, *p* = 0.01, and *p* = 0.015, respectively). The decision tree model showed that the first indicator of deep-seated infection was a D-dimer level above 1.15 mg/L, followed by a fibrinogen level above 334 mg/dL. **Conclusions**: Deep-seated *S. aureus* infections are more frequently associated with community-acquired cases, persistent hemoculture positivity, and methicillin-susceptible *Staphylococcus aureus* (MSSA) strains. Additionally, elevated D-dimer and fibrinogen levels may serve as valuable markers for identifying deep-seated infections in pediatric patients.

## 1. Introduction

*Staphylococcus aureus* is a leading cause of both community-associated (CA) and healthcare-associated (HA) invasive infections. According to The Lancet’s global report, *S*. *aureus* is identified as one of the top five pathogens responsible for infection-related deaths [1,2]. *S*. *aureus* may cause a variety of localized or invasive suppurative infections and toxin-mediated disorders. It is also both an integral part of the human flora and a common culprit in healthcare-associated infections related to catheters or shunts, as it easily attaches to prosthetic materials through surface molecules [3]. In the spectrum of *S. aureus* strains, methicillin resistance serves as a pivotal factor. Methicillin-sensitive *S. aureus* strains are defined as MSSA, whereas MRSA refers to those that exhibit resistance to methicillin. Previously identified as the causative agent of HA infections, MRSA has been responsible for CA infections since the early 2000s [4]. With its highly virulent and destructive microbiological features, invasive and fatal infections such as pyomyositis, deep tissue abscesses, osteoarticular infections, or septic pulmonary emboli remain significant clinical concerns [5]. All these complications can be defined as deep-seated infections. Sometimes, it is challenging to diagnose deep-seated infections, which can potentially lead to fatal outcomes due to treatment delays. Therefore, rapid diagnosis and initiation of appropriate treatment are crucial [6]. Given the propensity of deep-seated infections to induce thrombotic and inflammatory responses, there is increasing interest in identifying biomarkers that may support early detection and risk stratification [6,7]. One such marker is D-dimer, a fibrin degradation product commonly used in the evaluation of thrombotic conditions. However, it is a non-specific marker that may also be elevated in various clinical settings, including malignancy, trauma, and systemic inflammation [8]. Given its potential role in infection-related thrombosis, especially in deep-seated infections, D-dimer may be a useful diagnostic marker in pediatric *S. aureus* infections and warrants further investigation. This study investigates deep-seated *S. aureus* infections in children, focusing on their clinical presentation and the role of D-dimer and other biochemical parameters.

## 2. Materials and Methods

### 2.1. Patients

All children between 1 month and 18 years who had *S. aureus* growth in blood, pus, or joint fluid culture between 1 January 2018, and 1 June 2024, were included in this study. Necessary approval for this study was previously obtained from the Ethics Committee of our institution (date: 6 August 2024, number: 1059455). Children who had *S. aureus* growth in bronchoalveolar lavage, urine culture, or cerebrospinal fluid were excluded.

### 2.2. Retrospective Study

The following demographic and clinical data were retrospectively collected from the hospital’s electronic medical recording system: age, gender, underlying medical condition, co-infections, symptoms, deep infectious focus involvement, number of positive hemocultures, antimicrobial susceptibility profile, type, and duration of treatment. Patients were categorized into two groups, one with deep-seated infections and the remaining group. Patients were also classified into subgroups based on both the type of infection (community or healthcare-associated) and their age distribution (1–60, 61–144, 145–216 months). Deep muscle abscesses, arthritis, osteomyelitis, pulmonary involvement, and endocarditis are classified as deep-seated infections. Healthcare-associated infections are defined as infections with a positive culture obtained after 48 h of hospital admission or within 48 h of hospital discharge. *S. aureus* bacteremia (SAB) was defined as the presence of at least one blood culture that grew *S. aureus*. Blood cultures were obtained using standard techniques and incubated in the BACTEC blood culture system (Becton Dickinson, Franklin Lakes, NJ, USA). Positive cultures were subcultured on sheep blood agar and chocolate agar and incubated at 35–37 °C in aerobic conditions. Identification of *S. aureus* was based on colony morphology, Gram staining, catalase, and coagulase positivity. Final species confirmation was performed using a MALDI-TOF. Methicillin resistance was evaluated using the disc diffusion method with 30 µg cefoxitin discs on Mueller–Hinton agar plates. The zone diameters were interpreted according to the European Committee on Antimicrobial Susceptibility Testing (EUCAST) guidelines. Isolates showing resistance to cefoxitin were classified as methicillin-resistant *S. aureus* (MRSA). Quality control strains recommended by EUCAST were included in each batch of testing. Complete blood count parameters were measured using an automated hematology analyzer, Cell-Dyn 3700 (Abbott Laboratories, Chicago, IL, USA). Biochemical markers, including C-reactive protein (CRP), procalcitonin, creatinine, albumin, aspartate aminotransferase, and alanine aminotransferase, were analyzed using a routine clinical chemistry analyzer (Roche, Hitachi, Basel, Switzerland) in accordance with the manufacturer’s protocols and standard laboratory procedures.

### 2.3. Statistical Analysis

Data were analyzed using the Statistical Package for the Social Sciences (SPSS, version 26). Continuous data are displayed as mean (standard deviation) and median (minimum–maximum). Categorical data were illustrated as numbers and percentages. The chi-squared test was used to test an association between study groups and categorical variables. The Shapiro–Wilk normality test was used to assess the normal distribution of the numerical variables. Based on the results, the Mann–Whitney U test was employed to compare the single-measurement numerical variables of patients between the groups. Comparison between three different age groups was evaluated through pairwise comparisons, with Bonferroni correction (*p* < 0.0167). In addition, binary logistic regression analysis was performed to evaluate the predictive performance of biochemical markers for deep-seated infections. Receiver operating characteristic (ROC) curve analysis was performed to assess the diagnostic performance for the diagnosis of deep-seated infections. The area under the curve (AUC) was calculated to quantify the test’s overall ability to discriminate between the groups. Furthermore, decision tree analyses using Classification and Regression Tree (CRT) algorithms were conducted to identify the most proper predictors for deep tissue invasion. A *p*-value < 0.05 was assessed as statistically significant.

## 3. Results

**Figure 1:**
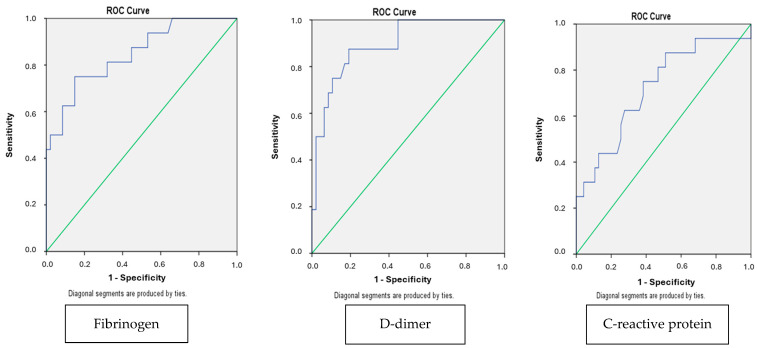
ROC curve demonstrating the diagnostic performance of fibrinogen, D-dimer, and CRP. In each ROC curve, the blue line represents the ROC curve itself, showing the relationship between sensitivity and 1-specificity. The green diagonal line indicates the line of no-discrimination (AUC = 0.5), representing a random classifier. These are standard elements of ROC plots.

**Figure 2:**
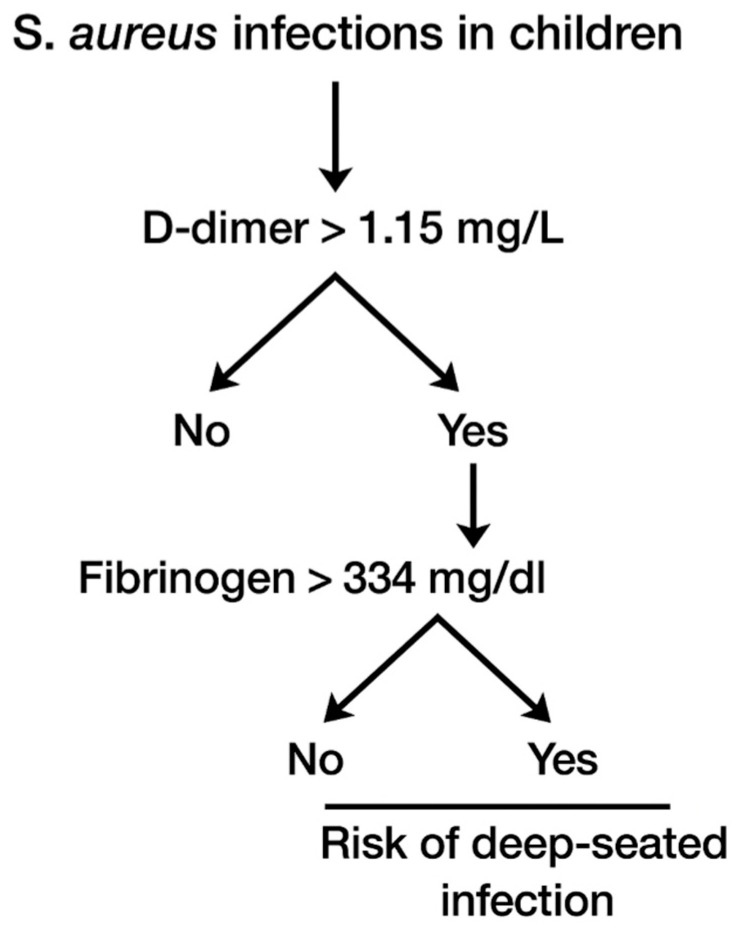
Decision tree analysis for deep-seated infections in *S. aureus*.

During the study period, 61 patients with *S*. *aureus* growth in culture, with a median age of 55 months (2–120 months), were included. A total of 32 (52.4%) children were female. A total of 40 (65.5%) children had *S*. *aureus* growth in hemoculture and were defined as having *S*. *aureus* bacteremia (SAB). Twenty-five percent of the children had deep-seated infections. Joint/bone infection was detected in 10 (16.3%) patients, while muscle and pulmonary involvement was found in 6 (9.8%) and 5 (8.1%) patients, respectively. The community-associated infection rate was 47.5%. Methicillin resistance rate was found in 17.2% of community-associated infections and 32.7% of all patients. When comparing MRSA and MSSA groups, community-acquired infections were significantly more common in the MSSA group (*p* = 0.021), as was the frequency of deep tissue invasion (*p* = 0.006). No other parameters showed a statistically significant difference between the groups. Prolonged hemoculture positivity exceeding 72 h was reported in 11.4% of all children. When evaluating the differences between deep-seated infections and the remaining groups, the CA infection rate, hemoculture positivity over 72 h, and methicillin-sensitive species were statistically significantly more common in the deep-seated infection group (*p* < 0.01) (Table 1). C-reactive protein, sedimentation rate, D-dimer, and fibrinogen values were statistically significantly higher in the deep-seated infection group (Table 2). ROC analysis demonstrated good diagnostic performance for D-dimer (AUC: 0.872, 95% CI: 0.784–0.960) and fibrinogen (AUC: 0.837, 95% CI: 0.728–0.946) in predicting deep-seated infections (Figure 1). C-reactive protein showed a lower, yet acceptable, diagnostic value (AUC: 0.721). Optimal cut-off values were 1.02 mg/L for D-dimer (sensitivity, 75%; specificity, 87.2%) and 334 mg/dL for fibrinogen (sensitivity, 81.3%; specificity, 78.7%). The decision tree model for deep-seated infection involvement (Figure 2) identified D-dimer levels greater than 1.15 mg/L as the first step, followed by fibrinogen levels greater than 334 mg/dL. The model yielded an overall accuracy of 88.9%, with a sensitivity of 62.5% and a specificity of 97.9%. Comparisons between the HA and CA infection groups were summarized in Table 3. Evaluation of patients by age group is presented in Table 4. Although some variables showed statistically significant differences in terms of age, no significant results were observed in pairwise comparisons after the Bonferroni correction.

## 4. Discussion

*Staphylococcus aureus* is one of the most important causes of community-associated skin, soft tissue, bone, and deep-seated infections, as well as nosocomial infections. In our study, we aimed to evaluate the various clinical manifestations of *S*. *aureus* and identify risk factors for deep tissue involvement, as the diagnosis and treatment of deep-seated infections often present significant challenges. Considering the differences between CA and HA infections, it is important to highlight that deep-seated infections predominantly occur within the CA group in our study (*p* < 0.01). Late hospital admission due to subtle symptoms and related delayed initiation of antibiotherapy may explain these severe clinical outcomes. Similar to our data, Suryati et al. [9] reported that bone and joint infections were significantly higher in the community-associated group than in the nosocomial group in their study.

Additionally, hemoculture positivity greater than 72 h was found to be related to deep-seated infections (*p* < 0.01). Similar to us, Ross et al. [6] found hemoculture positivity over 24 h associated with unsuspected deep-seated infections. Also, Jensen emphasized the relationship between persistent bacteremia and deep tissue invasion [7].

Studies investigating metastatic complications of *S*. *aureus* in different age groups revealed distinct outcomes [10,11,12]. For example, chronic renal or hepatic diseases, alcohol, and IV drug use were found to be the most common risk factors for metastatic *S*. *aureus* complications in adults with SAB. In contrast, congenital heart diseases in two patients and a history of trauma (14.7%) were the main risk factors in our study. At the same time, none of the children had an immune deficiency, a history of IV drug use, or renal or hepatic insufficiency in our study. Moreover, infective endocarditis is much more common in adults with metastatic complications of *S*. *aureus* [11]. It could be attributed to common intravenous drug use and related right-sided infective endocarditis in adults. Therefore, while routine echocardiography is recommended for adults with SAB, it is controversial in children with *S*. *aureus* bacteremia [9,12]. For children with *S*. *aureus* bacteremia, it may be reasonable to recommend echocardiography for those with prolonged fever, an unstable clinical condition, or an unknown source of infection rather than performing echocardiography on every child with SAB.

In another large-scale study on *S*. *aureus* infections in children, Ross et al. reported 3.7% of unsuspected deep-focus infections in 298 children diagnosed with SAB [6]. Differently, all children with deep-seated infections were symptomatic in our study. In our study, 40 patients with SAB were analyzed, and 25% of them were diagnosed with a deep-seated infection. Among them, 60% required ICU admission, mainly because of respiratory distress related to pulmonary involvement of *S*. *aureus*. As is known, *S*. *aureus* is a highly virulent pathogen known for causing destructive lung damage both in previously healthy children and those with chronic diseases [13]. A total of five children in our cohort were diagnosed with septic pulmonary emboli (SPEs) (Table 5). All children with SPEs had MSSA growth in hemoculture, and all of them had CA infections. The pathogenesis of SPEs involves the spread of a pathogen from an infected focus to the lungs via venous circulation in the presence of thrombosis. In addition, *S. aureus* is a risk factor on its own for thrombosis due to endothelial damage related to its toxins and virulence factors, even in the absence of infective endocarditis [14]. Only one child in these five cases with SPEs had an infective endocarditis diagnosis in our study. Pyomyositis and arthritis were the other underlying reasons for SPEs.

**Figure 3:**
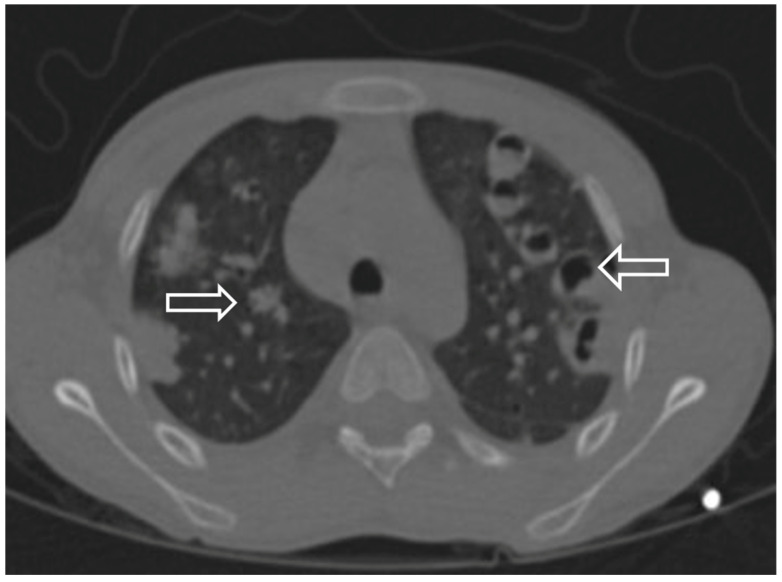
Contrast-enhanced chest computed tomography of a pediatric patient with septic pulmonary embolism showing nodular infiltrates and cavitations.

Case 5 was a 9-year-old boy with a history of neck trauma and jugular vein thrombosis. Goswami et al. [14] reported 40 adult patients diagnosed with SPEs, most of whom were related to *S*. *aureus* bacteremia, and 27% of them had infective endocarditis. They also emphasized nodular lesions in the peripheral areas of the lungs, cavitations, ground-glass opacities, and reverse halo signs as the most common findings on computed tomography. Similarly, nodular infiltration, cavitation, reverse halo sign, effusion, and ground glass opacity were present in our patients with SPEs (Figure 3). All children with SPEs received systemic antibiotherapy and were followed up in the intensive care unit. A total of two patients required chest tube placement because of pleural effusion and pneumothorax (Cases 1 and 2). Clinical improvement was observed in all patients with SPEs, and no mortality was observed.

**Figure 4:**
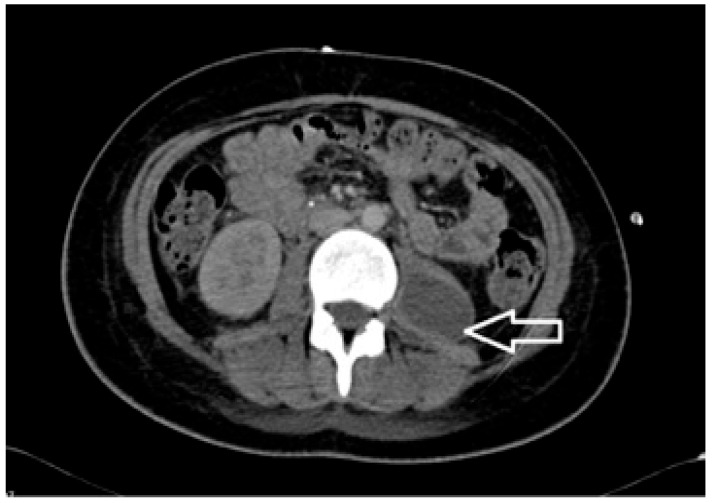
Contrast-enhanced computed tomography of an adolescent girl with pyomyositis demonstrating involvement of multiple muscle groups, including paravertebral muscles.

Regarding another deep-seated complication, pyomyositis—a rare acute bacterial infection affecting skeletal muscles—is primarily caused by *S. aureus* in children [15]. In our research, six children were diagnosed with pyomyositis, each with MSSA growth on blood/pus culture (Table 6). The median age was 160 months in the pyomyositis group (ranging from 108 to 204 months). In line with our results, pyomyositis is commonly reported in adolescents in the literature [15]. Pyomyositis typically occurs in sites that are easily traumatized, such as the arm, leg, chest, or abdominal wall. However, cases of intra-abdominal pyomyositis caused by several pathogens have also been reported [15,16]. All of the patients had pyomyositis in the iliopsoas muscle. Unlike the other patients, only an adolescent girl (Case 1, Table 6) had multiple muscle involvement (bilateral thoracic paravertebral, psoas, iliopsoas, pectoral, pelvic, obturator, and gluteal muscles) along with multiorgan failure and sepsis (Figure 4).

Among all patients with deep tissue infections in our study, this patient had the most challenging clinical condition and required a prolonged stay in the intensive care unit. In addition to sepsis and pyomyositis, she had pericardial and pleural effusions and a skin rash as well. She made a full recovery after receiving antibiotherapy and supportive care. If we evaluate the pathogenesis of pyomyositis, bacteremia that seeds damaged muscles is considered a significant cause in some cases. Vigorous exercise and trauma are also considered to be underlying conditions for pyomyositis, even in previously healthy children [17]. Considering risk factors, only one of the patients in our study had a history of trauma, and another one had morbid obesity. Maravelas et al. reported obesity as a risk factor for pyomyositis in their report [18]. Another risk factor for pyomyositis is primary or secondary immunodeficiency [19]. Conversely, immunophenotypic analyses, immunoglobulin values, and the nitroblue tetrazolium test results of all children in our study were within normal ranges. Also, anti-HIV was negative in all of the children. Consistent with our results, the literature reports numerous pediatric cases of pyomyositis in previously healthy children [20,21]. Another critical aspect of pyomyositis is the risk of bone or joint involvement via dissemination. Therefore, 83% of our patients had been diagnosed with pelvic osteomyelitis during follow-up and received long-term antibiotherapy.

Investigating the reasons behind deep-seated infections, the primary mechanism in the progression from bacteremia to secondary infection appears to depend on various virulence factors and toxins of *S*. *aureus,* which are responsible for adhesion, invasion, platelet activation, and immune evasion. For example, Panton–Valentine leukocidin (PVL) is a toxin produced by *S*. *aureus,* which is particularly associated with skin and soft tissue infections and is recognized as a virulence factor for severe infections [22]. Unfortunately, we did not have the opportunity to examine this toxin in our study.

Previous pediatric studies report a rate of methicillin resistance ranging between 10 and 29% [12,23,24]. The total methicillin resistance rate was 32.7% in our research. It is essential to emphasize the statistically significant correlation between methicillin-sensitive *S. aureus* infections and deep tissue invasion and severe complications in our study. In contrast, a large-scale meta-analysis found MRSA bacteremia to be associated with higher mortality [25]. In line with this outcome, it is widely believed that MRSA infections tend to be more complicated and have a worse outcome, as noted in several reports [24,25]. Several factors, in addition to methicillin sensitivity, such as PVL, may contribute to the varied outcomes observed in our research.

When evaluating laboratory parameters in the deep-seated infection group, CRP, sedimentation rate, fibrinogen, and D-dimer values are higher in cases of deep tissue invasion (Table 2). High CRP and sedimentation values are not surprising, considering the severe inflammatory process. Furthermore, our findings suggest that deep-seated infections may also affect the coagulation system, as shown by the high levels of D-dimer and fibrinogen. D-dimer, one of the key fibrin degradation products, is frequently elevated in various clinical conditions, including thromboembolism, inflammation, sepsis, malignancies, and trauma. Moreover, fibrinogen is a glycoprotein involved in coagulation, inflammation, immune response, and tissue repair. Elevated fibrinogen levels indicate systemic inflammation and activation of the coagulation system [26].

Several studies have explored the relationship between infections and coagulation parameters. For example, Li et al. reported the diagnostic value of CRP, procalcitonin, D-dimer, and total leukocyte count in differentiating Gram-positive and Gram-negative bloodstream infections [27]. Additionally, previous research has explored the relationship between elevated D-dimer levels and conditions such as prosthetic joint infections or infective endocarditis [28,29]. However, to our knowledge, no studies have specifically examined the relationship between D-dimer and fibrinogen levels and deep tissue involvement in *Staphylococcus aureus* infections in childhood. In our study, the combination of fibrinogen levels above 334 mg/dL and D-dimer levels above 1.15 mg/L was identified as a risk factor for deep-seated infections. This outcome may be a result of increased inflammation during deep tissue invasion and the subsequent occurrence of thrombosis.

The limited number of patients and the retrospective design were the main limitations of our study. Although a statistically significant decision tree was established for D-dimer and fibrinogen in predicting deep-seated infections, the small sample size and the fact that a wide range of clinical conditions can influence both markers limit the diagnostic certainty of the model. Therefore, the decision tree should be interpreted as a supportive tool that may aid clinical judgment rather than provide definitive diagnostic criteria. The strengths of our study include the comparative evaluation of deep-seated *S. aureus* infections and severe complications in children, as well as the novel use of D-dimer and fibrinogen as potential markers of invasive disease. Even though the decision tree is limited by the small number of patients and the fact that the markers are not specific, it can still be a helpful tool in clinical evaluation.

In conclusion, this study examined the clinical features of *S. aureus* in community-associated infections and healthcare settings. Our results highlight a prominent tendency towards deep-seated metastatic complications in community-associated *S*. *aureus* bacteremia. Additionally, high levels of D-dimer and fibrinogen have been identified as independent risk factors for deep tissue involvement. Community-associated *S. aureus* bacteremia requires careful follow-up for potential deep-seated involvement and severe complications, especially septic pulmonary emboli. Future studies with larger, prospective pediatric cohorts are needed to validate the diagnostic cut-offs of D-dimer and fibrinogen combined with new biomarkers to improve the early prediction of deep-seated *Staphylococcus aureus* infections. Furthermore, specialized microbiological evaluations, such as testing for Panton–Valentine leukocidin, may help clarify the risk factors associated with invasive disease.

## Figures and Tables

**Table 1:** Comparison between deep-seated infections and the remaining groups.

Variables	Groups				*p*-Value
	Deep-Seated Infection (*n* = 14)		Other (*n* = 47)		
	N	%	N	%	
Gender					0.187
Male	9	64.3	20	42.5	
Female	5	35.7	27	57.5	
Community/hospital-associated infection					<0.01
Community	14	100	16	34	
Hospital	0	0	31	66	
Methicillin resistance					<0.01
Yes	0	0	20	42.5	
No	14	100	27	57.5	
Hemoculture positive >72 h					<0.01
Yes	7	50	0	0	
No	7	50	46	100	
Fever					0.923
Yes	9	64.3	23	48.9	
No	5	35.7	24	51.1	
Intensive care unit admission					0.957
Yes	6	42.8	17	36.1	
No	8	57.2	30	63.9	

**Table 2:** Comparison of laboratory markers between deep-seated infections and the remaining group.

Variables	Mean Values of Laboratory Values Groups		*p*-Value
	Deep-Seated Infection (*n* = 14)	Other (*n* = 47)	
Hemoglobin (g/dL)	11.252 ± 15.096	10.963 ± 17.378	0.562
Total leucocyte count (×10^9^/L)	13.465 ± 7.687	11.470 ± 5.449	0.465
Neutrophil count (×10^9^/L)	9.205 ± 6.949	7.199 ± 5.362	0.246
Platelet count (×10^9^/L)	363.647 ± 22.420	343.434 ± 155.350	0.190
D-dimer (mg/L)	18.630 ± 25.653	2.317 ± 3.870	0.010
Fibrinogen (mg/dL)	492.300 ± 196.364	229.0 ± 118.597	0.015
C-reactive protein (mg/L)	134.634 ± 155.305	45.214 ± 64.803	0.020
Procalcitonin (ng/dL)	20.132 ± 38.98	4.949 ± 17.152	0.789
Sedimentation rate (mm/h)	56.633 ± 39.713	24 ± 22.242	0.018
Creatinine (mg/dL)	0.574 ± 0.651	3.863 ± 0.613	0.042
Albumin (g/dL)	3.657 ± 0.766	3.863 ± 0.613	0.315
Aspartate aminotransferase (IU/L)	42.570 ±26.997	53.130 ± 57.634	0.667
Alanine aminotransferase (IU/L)	33.052 ± 24.712	42.169 ± 55.612	0.378

**Table 3:** Comparison between community- and healthcare-associated infections.

Variables	Groups				*p*-Value
	Community-Associated (*n* = 29)		Healthcare-Associated (*n* = 32)		
	N	%	N	%	
Gender					0.102
Male	17	58.6	12	37.5	
Female	12	41.4	20	62.5	
Deep focus involvement					<0.01
Yes	14	48.2	0	0	
No	15	51.8	32	100	
Methicillin resistance					0.21
Yes	5	17.2	15	46.9	
No	24	82.8	17	53.1	
Hemoculture positive >72 h					0.011
Yes	7	24.1	0	0	
No	22	75.9	31	100	
Fever					0.235
Yes	14	48.2	18	62.1	
No	15	51.8	11	37.9	
Intensive care unit admission					0.024
Yes	7	24.1	16	50	
No	22	75.9	16	50	

**Table 4:** Comparison between age groups (months).

Variables	1–60 (*n* = 31)	61–144 (*n* = 16)	145–216 (*n* = 14)	*p*-Value
	(*n*,%)	(*n*,%)	(*n*,%)	
Gender				0.861
Male	15 (48.3%)	7 (43.7%)	7 (50%)	
Female	16 (51.7)	9 (56.3%)	7 (50%)	
Deep focus involvement				0.035
Yes	3 (9.6%)	5 (31.2%)	6 (42.8%)	
No	28 (90.4%)	11 (68.8%)	8 (57.2%)	
Methicillin resistance				0.368
Yes	12 (38.7%)	5 (31.2%)	2 (14.2%)	
No	19 (61.3%)	11 (68.8%)	12 (85.8%)	
Hemoculture positive >72 h				0.018
Yes	0 (0%)	4 (25%)	3 (21.4%)	
No	31 (100%)	12 (75%)	11 (78.6%)	
Fever				0.637
Yes	14 (45.1%)	10 (62.5%)	8 (57.1%)	
No	17 (54.9%)	6 (37.5%)	6 (42.9%)	
Intensive care unit admission				0.868
Yes	12 (38.7%)	5 (31.2%)	6 (42.8%)	
No	19 (61.3%)	11 (68.8%)	8 (57.2%)	

**Table 5:** Clinical features of children diagnosed with septic pulmonary involvement.

	Case 1	Case 2	Case 3	Case 4	Case 5
Age	15 years	17 years	11 years	15 years	9 years
Gender	Female	Male	Female	Female	Male
Comorbidities	No	No	Atrial septal defect	No	No
Symptoms	Respiratory distress	Respiratory distress	Respiratory distress	Respiratory distress	Respiratory distress
Extrapulmonary source	Pyomyositis Arthritis	Pyomyositis Osteomyelitis Arthritis	Endocarditis	Arthritis	Arthritis
Thrombosis	No	Yes	No	No	Yes
CT	Effusion	Nodul Cavitation Reverse halo Pneumothorax	Nodul Cavitation Reverse halo	Nodul Ground glass opacity	Nodul Cavitation Reverse halo sign
Trauma	No	Yes	No	Yes	Yes

**Table 6:** Clinical features of children diagnosed with pyomyositis.

	Case 1	Case 2	Case 3	Case 4	Case 5	Case 6
Age	15 years	13 years	10 years	17 years	17 years	15 years
Gender	Female	Female	Female	Male	Male	Male
Trauma	No	Yes	No	Yes	No	No
Muscles	Gluteus, Iliopsoas Pectoral, Paravertebral, Obturator, Iliac	Iliopsoas	Iliopsoas	Iliopsoas, Gluteus	Iliopsoas	Iliopsoas
Symptoms	Fever, respiratory distress, pain	Pain	Pain	Fever, respiratory distress, pain	Pain	Pain
Arthritis/OM	Yes	Yes	Yes	Yes	Yes	No

## Data Availability

The data supporting the findings of this study are not publicly available due to privacy restrictions.

## Abbreviations

The following abbreviations are used in this manuscript:

CRP	C-reactive protein
SAB	Staphylococcus aureus bacteremia
SPEs	Septic pulmonary emboli
CA	Community-associated
HA	Healthcare-associated
MSSA	Methicillin-sensitive *Staphylococcus aureus*
MRSA	Methicillin-resistant *Staphylococcus aureus*
